# Rsearch progress on the relationship between aging and microbiota in sarcopenia

**DOI:** 10.3389/fmed.2025.1549733

**Published:** 2025-08-07

**Authors:** Jing-Ping Cheng, Xiang Chen, Jia-Xin Chen

**Affiliations:** ^1^Department of Gerontology, CR & WISCO General Hospital Affiliated to Wuhan University of Science and Technology, Wuhan, Hubei, China; ^2^School of Medicine, Medical College, Wuhan University of Science and Technology, Wuhan, Hubei, China

**Keywords:** sarcopenia, gut microbiota, immunosenescence, T cell senescence, intestinal dysbacteria

## Abstract

Sarcopenia is an age-related skeletal muscle disease associated with adverse outcomes such as falls, decreased function, frailty, and death, and is a significant global public health problem that impairs the functioning of individuals. Aging is intensifying, the number of people with sarcopenia is increasing, and there are currently no specific treatment drugs for sarcopenia. The clinical pathogenesis of sarcopenia is extremely complex, and the underlying mechanism of immunosenescence and dysbiosis associated with aging on sarcopenia is not well studied, and they are also potential therapeutic targets for sarcopenia. This review mainly discusses the relationship between sarcopenia from the perspective of intestinal microbiota dysbiosis and T cell changes in immunosenescence, and looks for promising targets for diagnosis or intervention of sarcopenia in the future, hoping to achieve early detection, early diagnosis and early treatment of sarcopenia and prolong the life span of healthy aging.

## 1 Introduction

Sarcopenia, initially coined by researcher Irwin Rosenberg in 1989, is characterized as the age-related decline in lean body mass that impacts physical function and nutritional status (1). The European Working Group on Sarcopenia in Older People (EWGSOP) has promulgated a widely adopted definition of sarcopenia, which is globally acknowledged: It denotes a geriatric syndrome characterized by age-related diminution in muscle mass, muscle strength, and/or physical function. This definition is recommended for sustained utilization in the 2023 Chinese expert consensus on the prevention and management of sarcopenia in the elderly (2, 3).

Sarcopenia is associated with adverse outcomes such as falls, functional decline, frailty, and mortality. This includes an elevated risk of falls and hospitalizations, diminished capacity for activities of daily living, increased functional impairment, heightened incidence of multisystem diseases (cardiovascular (4), respiratory (5), neurological (6), etc.), reduced quality of life, loss of independence or necessity for long-term care placement, and even death (7). Consequently, this has significantly augmented the burden on individuals and society (8–10).

The pathogenesis of sarcopenia is highly complex. The investigation into immune senescence induced by aging, dysbiosis, and sarcopenia remains incomplete, and there is a lack of precise understanding regarding the potential mechanism of sarcopenia. This article primarily summarizes and analyzes the correlation between intestinal flora imbalance and T-cell alterations in immune senescence and sarcopenia. In the future, further scientific research is imperative to gain a profound understanding of the potential mechanisms linking immune senescence, intestinal flora imbalance, and sarcopenia, as well as to identify early diagnostic targets and therapeutic drugs for sarcopenia.

## 2 Contemporary epidemiological trends in sarcopenia

In 2016, the World Health Organization officially included sarcopenia as a separate and independent disease with distinct characteristics in the tenth edition of The International Statistical Classification of Diseases and Related Health Problems (ICD-10) code (2, 11). This recognition marks an important step in distinguishing sarcopenia as a treatable disease and increasing attention to it (11, 12). In recent years, there has been a substantial increase in the global population of individuals aged 60 years and older. According to the “Aging and Health” report released by the World Health Organization on October 1, 2022, it is projected that the number of people over 60 will rise from 1 billion in 2020 to 1.4 billion by 2030, and further to reach 2.1 billion by 2050. As aging becomes more prominent, the prevalence of sarcopenia is expected to increase concurrently. Currently, approximately 50 million individuals worldwide are estimated to be affected by sarcopenia, with this number anticipated to escalate to 500 million by the year 2050 (13).

Sarcopenia, a geriatric syndrome, is increasingly recognized as a prevalent yet frequently underdiagnosed health concern. Its prevalence among the elderly is widely acknowledged to be variable, ranging from 5 to 50%, influenced by factors such as gender, age, comorbidities, and diagnostic criteria (14). The data revealed that the prevalence of sarcopenia among foreign community residents was 11% in males and 9% in females. In nursing home residents, the prevalence of sarcopenia was 51% for both sexes. However, hospitalized patients exhibited a prevalence of 23% in males and 24% in females (14, 15). Notably, sarcopenia appears to be more prevalent among individuals from non-Asian regions compared to Asian counterparts. The most recent epidemiological survey on sarcopenia in China indicates that the prevalence of sarcopenia among elderly community dwellers is 12.9% in males and 11.2% in females. Among hospitalized elderly individuals, the prevalence stands at 29.7% for males and 23.0% for females (16).

The wide range of prevalence is due not only to differences in Settings and populations, but also to the diversity of diagnostic criteria used. For example, a review published in “Metabolism-clinical And Experimental” in 2023 stated that sarcopenia affects 10–16% of the global elderly (17). The prevalence of sarcopenia ranges from 18% in patients with diabetes to 66% in patients with unresectable esophageal cancer (17). In Brazil, according to the 2015–2016 data, the critical point to define low muscle strength is <36 kg for men/<23 kg for women, and the prevalence of sarcopenia is 13.8% and the prevalence of possible sarcopenia is 40.1% (18). In Italy, a cross-sectional analysis of 655 participants in a multicenter observational study of older adults in 12 acute hospital wards concluded that the overall prevalence of sarcopenia was 34.7% and increased dramatically with age (19). In Taiwan, a 2021 study counted 173 participants aged ≥65 years from day care centers in northern Taiwan and found that the prevalence of sarcopenia was 50.9%, with probable sarcopenia 47.4% and normal 1.7%. In Europe and the United States, the prevalence of sarcopenia with different diagnostic criteria ranges from 4.6 to 43% in the community and from 23 to 68% in clinical Settings (20).

## 3 Immunosenescence and sarcopenia

The pathogenesis of sarcopenia is exceedingly complex, particularly with regard to age-related mechanisms contributing to its onset, including inflammation, immune dysfunction, cell senescence, anabolic resistance, abnormal skeletal muscle repair, intestinal microbiota imbalance, reduced physical activity levels and increased oxidative stress (21, 22). Our understanding of age-related immune senescence and dysbiosis and their association with sarcopenia remains incomplete. Moreover, the underlying mechanism of sarcopenia lacks precise comprehension. Currently, there are no effective pharmaceutical treatments for sarcopenia. Some researchers have proposed that immune aging and intestinal flora may regulate the development of sarcopenia and thus represent potential therapeutic targets worthy of further investigation (23, 24).

### 3.1 Immunosenescence

As a key pathogenic factor of sarcopenia, immunosenescence has a complex mechanism. Immunosenescence is the dysfunction and decline of the immune system that occurs during aging (25), involving innate and adaptive immunity. The alterations in the adaptive immune system (including B cells, CD4^+^T cells, and CD8^+^T cells) are particularly significant, with T cell changes being the most prominent (23, 26). Over the past few decades, extensive research has been conducted on immunosenescence in human and animal models. This process is marked by thymic degeneration, accumulation of senescent T cells, impaired function of innate immune cells (such as natural killer cells, macrophages, and neutrophils), as well as compromised maintenance and functional response of lymphocytes. Age-related alterations in skeletal muscle immune function have also been observed (27), with thymic degeneration playing a predominant role in immunosenescence. It is extensively documented that the thymus serves as a primary lymphoid organ responsible for T cell differentiation, development, and maturation (26).

The alterations in T cells are most prominent during immune aging, and these changes may not only diminish normal immune function but also trigger an inflammatory tendency (28), which is considered the primary cause for the heightened frequency and severity of diseases and infections in the elderly (29). Furthermore, it is also a significant factor influencing the onset and progression of sarcopenia.

In both CD4 and CD8 cells, naive T cells’ proportion and absolute number progressively decline with age. This phenomenon is attributed to thymic degeneration and inadequate homeostatic proliferation (30). Given these characteristics, it is imperative to consider interventions to enhance T cell production and restore thymic function to prevent future immune senescence.

Regulatory T cells (Tregs), a specialized subset of CD4 + T cells that emigrate from the thymus and are generated through peripheral conversion of conventional CD4 + T cells, play a crucial role in maintaining central and peripheral immune tolerance and are distributed throughout various tissues and mucosal surfaces, including the intestine (31). In addition to regulating inflammation, Tregs can also modulate muscle regeneration (31, 32). Dysregulated Tregs cell homeostasis has long been observed in aged mice and the elderly (33).

### 3.2 Immunosenescence and sarcopenia

At present, the evidence that immunity is related to sarcopenia is increasing year by year. Studies have confirmed that immunosenescence plays a role in age-related muscle atrophy, muscle fiber denervation, and reduced regeneration after injury (34). Previous reports have suggested a potential link or interaction between sarcopenia and immunosenescence through skeletal muscle (35), with immunosenescence exacerbating the effects of muscle wasting during aging, thus serving as a key factor in the development of sarcopenia.

Prior investigations have established that immunosenescence is characterized by an imbalance of immune cells and their subtypes, as well as the production of aging-related secretory phenotypic factors (26), which can attenuate muscle protein synthesis and enhance muscle protein breakdown (35, 36), ultimately leading to sarcopenia. Furthermore, immunosenescence may also contribute to the age-related decline in regeneration following muscle atrophy (34) and could potentially hinder the repair of skeletal muscle injury to some extent (37). The intricate relationship between changes in T cells during immune aging and sarcopenia warrants further investigation. Several potential mechanisms are outlined below.

Firstly, T cells not only release perforin and granzyme B cytotoxic particles but CD28^−^ T cells also have the capacity to produce inflammatory cytokines such as TNF-*α* and IFN-*γ* (38). The elevated inflammatory state can lead to the occurrence of sarcopenia. It has been reported that the levels of biomarkers of the immune system, such as C-reactive protein (CRP), Tumor necrosis factor-*α* (TNF-α), reactive oxygen species (ROS), and interferon-*α*, increase slightly with age (39). Studies have found that TNF-α activates signal transduction by binding to its cell surface receptors, TNFR1 and TNFR2. By binding to TNFR1, Tnf-α increases the recruitment and activation of inflammatory cells, promotes the release of inflammatory factors, and ultimately exacerbates the chronic inflammatory state (40). Excessive production of these cytokines can accelerate the deterioration of skeletal muscle fiber diameter and protein content. They can also bind to corresponding receptors, initiating nuclear apoptosis in skeletal muscle through a series of reactions (36), thereby impacting muscle regeneration. Furthermore, they can promote metabolic breakdown in skeletal muscle leading to proteolysis and apoptosis of muscle cells (41). It has been shown that TNF-*α* disrupts the PI3 kinase /PKB signaling pathway, leading to L6 myotubes atrophy and cell death. In addition, it also directly acts on skeletal muscle cells by activating nuclear factor kappa light chain enhancer (NF-κB) and MAPK signaling pathways in activated B cells, promoting muscle protein breakdown and inhibiting muscle synthesis (40). For example, elevated expression of TNF-*α* in muscles is associated with muscular weakness; loss of muscle strength and mass is linked to worsening sarcopenia which results in degradation of skeletal muscle protein content and subsequent fatigue along with limitations on physical activity (42). TNF-*α* is implicated not only in the pathogenesis of sarcopenia but also in the downregulation of CD28 expression on T cells, leading to the accumulation of CD28—T lymphocytes, a hallmark of immune aging that contributes to a vicious cycle of sarcopenia (36). A genetic mouse model has shown that targeting NF-κB is critical to preventing skeletal muscle loss by genetically expressing a constitutively active endogenous inhibitor of NF-κB (IkappaBalpha, IκB) mutant to achieve muscle-specific inhibition of NF-κb. Denervation-induced atrophy was significantly reduced (40, 43). Therefore, the improvement or regulation of skeletal muscle function through TNF-*α* signaling pathway should be further studied in the future.

Immunosenescence results in a significant reduction in the number and function of satellite cells, as well as in the regenerative capacity of aging muscles (44, 45), and shifts satellite cells to a fibrogenic phenotype (46), thereby impairing muscle regeneration and leading to muscle atrophy. In response to the diminished capacity of T cells to regulate the function of satellite cells (skeletal muscle stem cells) during aging, relevant studies using T cell-deficient mice and activated splenic T cell mice have been conducted by some researchers. They have found that the adaptive immune response of T cells, releasing cytokines into damaged muscles, promotes the continuous proliferation of satellite cells. Additionally, aging may modify the function of muscle precursor cells induced by T cells and lead to sarcopenia (42, 47, 48). Studies have indicated that primary sarcopenia’s pathogenesis is linked to the disruption of satellite cell characteristics. A decrease in both number and function of satellite cells has also been observed in sarcopenia patients (49). Therefore, as crucial contributors to muscle growth, senescent T-cells will reduce their proliferative ability, subsequently impacting muscle regeneration and leading to sarcopenia. Henceforth, it is imperative for further research on interventions targeting T-cell influence on satellite cell function aimed at enhancing muscular proliferation and differentiation among older individuals as a means to prevent or reverse age-related muscle loss.

Secondly, the direct cytotoxic effect of T cells can compromise the structural integrity of muscle fibers (50). The accumulation of memory T cells, particularly CD8+, is also considered detrimental in older individuals (51, 52). It has been proposed that the modification decline of T cell phenotype from CD8^+^ to CD4^+^ during immune aging may be associated with the loss of muscle mass (38).

Thirdly, a certain proportion of Treg cells not only facilitate muscle repair by regulating double regulatory protein (a growth factor targeting muscle stem cells) and extending the proliferation cycle of satellite cells (53) but also suppress muscle inflammation, making them crucial for muscle repair and regeneration (54, 55). However, in comparison to young individuals, T cells (especially Treg cells) in older adults fail to release appropriate factors (such as produced growth factors and cytokines (33, 56, 57) necessary for satellite cell proliferation and differentiation) into the microenvironment. This leads to inadequate muscle recovery and age-related deficiencies in muscle size and function (33). Another study found an increased frequency of Tregs in the spleen in aged mice, but they were unable to migrate to and accumulate in the injured skeletal muscle. This may lie in the fact that Tregs in aged mice show reduced expression of genes for the chemokine receptors CXCR6 and CCR7 and the S1P1 receptor that controls lymphocyte efflux from lymphoid organs (45). Research has revealed that without stimulation from Tregs during immune senescence, muscle satellite cells tend to proliferate rather than differentiate, resulting in the accumulation of satellite cells and loss of muscle mass (58). Pharmacological intervention targeting Tregs could facilitate muscle satellite cell expansion and promote muscular repair, thereby enhancing overall muscle regeneration.

In summary, these studies suggest that an aging immune system, as well as the inability of aging T cells to accumulate in injured muscle, is a key cause of the impaired regenerative capacity of aging muscle.

## 4 Relationship between sarcopenia and gut microbiota dysbiosis

### 4.1 Gut microbiota

The gastrointestinal tract functions as the body’s primary immune organ. Research indicates that it can accommodate up to 70% of the body’s lymphocyte population and plays a crucial role in maintaining immune balance (59). The microbiota and the immune system have established a mutually beneficial relationship (60). Key metabolites, such as Short-Chain Fatty Acids (SCFAs) including acetate, butyrate, and propionic acid, permeate the intestinal epithelial barrier and interact with host cells to regulate immune responses and disease susceptibility (60). Additionally, age-related gut flora dysbiosis may increase susceptibility to various diseases, such as sarcopenia, inflammatory bowel disease, diabetes, and cardiovascular disease, by compromising intestinal barrier integrity and inducing immune dysfunction (61).

### 4.2 Aging/dysbiosis of gut microbiota

Research indicates that the gut microbiota undergoes age-related alterations (Dysbiosis of intestinal flora) in older individuals. This is characterized by a general decline in resilience of the gut microbiota after the age of 65, leading to significant changes in its overall composition due to factors such as medication and diseases (62, 63). These changes entail reduced species richness, decreased biodiversity, increased presence of opportunistic Gram-negative bacteria, and diminished representation of species with purported health-promoting functions. For instance, there is a decrease in the abundance of core symbionts like Bacteroides, Bifidobacterium, and short-chain fatty acid (SCFA) producers (e.g., *Faecalibacterium prausnitzii*, Eubacterium, Rozella and Ruminococcus) (64–66), while Proteobacteria and other opportunistic microorganisms (such as Fusobacterium, Paracbacteroides, and Ruminococcaceae), which are normally low in healthy young individuals, are expanded among the elderly (66, 67).

### 4.3 Dysbiosis in the gut microbiota contributes to immune system aging

The maintenance of intestinal homeostasis is intricately regulated by the activity of intestinal T cells. These T cells intricately secrete both anti-inflammatory and pro-inflammatory cytokines to ensure a balance in the self-renewal and differentiation of intestinal stem cells, thereby maintaining a high turnover rate of the intestinal epithelium (68). However, as individuals age, the control of intestinal homeostasis by T cells diminishes, leading to inflammatory pathology: proinflammatory cytokines can induce differentiation of intestinal stem cells and may result in damage to the intestinal barrier (69). Clark et al. demonstrated using the Drosophila model that with aging, dysfunction in the intestinal barrier and imbalance in gut flora can induce systemic immune activation through the JAK–STAT pathway, ultimately contributing to mortality (70). Additionally, an intricate germinal center response is coordinated among various cell types including steady-state T follicular helper (TFH) cells, T follicular regulatory (TFR) cells, TH17 cells, and Treg cells aimed at establishing a symbiotic relationship between host and microbe through secretion of microbiota-specific IgG and local high-affinity IgA by plasma cells. Due to the abnormal composition of T cells in the germinal center during aging, this reciprocal relationship is disrupted, resulting in perturbation of the intestinal microbial community (68, 71–73). This disruption manifests as intestinal dysbiosis caused by dysregulated T-cell response and may underlie aging-related inflammation. In summary, age or disease-induced imbalance of intestinal microbiota disrupts its composition and disturbs the equilibrium between microbiota and immune system. Consequently, immune tolerance to commensal bacteria is compromised, epithelial barrier function is impaired, intestinal permeability is enhanced, and there is an imbalance in activation between anti-inflammatory T regulatory lymphocytes and pro-inflammatory Th17 lymphocytes (60, 74, 75). As a result, systemic inflammation becomes chronically activated and continues to impact gut microbiota. Thus, a detrimental cycle forms between intestinal flora and the immune system (76). Furthermore, microbe-T cell interactions have emerged as a promising therapeutic target for age-related diseases (68).

The decrease in microbial diversity as individuals age results in a decline in the availability of short-chain fatty acids (SCFAs), such as acetate, butyrate, and propionate. This reduction facilitates uncontrolled bacterial proliferation, leading to inflammation and the onset of chronic diseases in older individuals (77). SCFAs are renowned for their pivotal role as signaling molecules in maintaining host metabolic and immune homeostasis (playing a crucial part in T cell homing (78)) and preserving intestinal barrier integrity (79, 80). Specifically concerning the intestinal barrier, butyrate acts as a distinctive energy source for colonic cells, regulating intestinal energy metabolism while suppressing colonic inflammation (81) and enhancing the intestinal barrier through the AMPK pathway (82). The protective impact of butyrate on the intestinal barrier is critical for preventing the translocation of microbe-associated molecular patterns (MAMPs) into the bloodstream and alleviating chronic inflammation in peripheral organs, including muscle (83). Regarding immune regulation, reduced microbial diversity heightens infection risk at distal mucosal sites like the lungs and affects host immune function (84). Co-housing young germ-free mice with old mice was found to elevate cytokine levels and impair macrophage function in young germ-free mice (85), indicating a causal relationship between age-related microbial dysbiosis and immune senescence for the first time. Another study confirmed that transferring gut microbiota from aged conventional mice to young germ-free mice promoted T-cell activation and small intestinal inflammation (86). In conclusion, the aging process leads to decreased SCFAs-producing microorganisms in the gut, such as *Faecalibacterium*, and a reduced availability of SCAFs. This has a detrimental impact on the integrity of the intestinal barrier, inflammation control, and immune function.

### 4.4 Imbalance in the gut microbiota and sarcopenia

Despite the anatomical separation between skeletal muscle and the gut, signals originating from the gut due to its interaction with the gut microbiome play a crucial role in connecting gut microbiota activity with skeletal muscle. The convergence of altered gut microbiota composition, physiological balance disruption, and muscle catabolic state induction suggest that the microbiota may directly or indirectly impact muscle mass status and regulation (87). Hence, the concept of a “gut-muscle axis” (i.e., the influence of gut microbiota and its interactions with the host gut on skeletal muscle metabolism and function) is particularly relevant in older sarcopenic adults (87). Increasing evidence has emphasized the pivotal role of the myenteric axis in sarcopenia.

Numerous studies have demonstrated an association between dysbiosis of gut microbiota and sarcopenia. For example, age-related increases in Firmicutes abundance and decreases in Bacteroidetes abundance observed in animal studies may be linked to reduced muscle mass and function. This unfavorable intestinal ecological environment could contribute to the development of sarcopenia. Conversely, a favorable intestinal ecological environment is conducive to preventing and treating sarcopenia (74). Animal studies have indicated that short-chain fatty acids (SCFAs), particularly butyrate, produced by gut microbes are beneficial for skeletal muscle mass (80, 88). It has been reported that acetate and propionate may be inversely correlated with muscle mass development (89). Unlike other SCFAs, butyrate exhibits histone deacetylase inhibitory activity, modulating DNA unwinding to regulate intestinal macrophages and promote peripheral Tregs cells, enhancing muscle differentiation and reducing muscle atrophy. However, with aging, there is a decrease in gut microbes producing SCFAs, which may be associated with sarcopenia (90). Professor Henderson’s research has unveiled a significant correlation between fecal butyrate and human skeletal muscle mass in sarcopenia. This study has identified butyrate as a biomarker associated with muscle mass in older adults. Furthermore, Professor Henderson has substantiated that elderly individuals with low muscle mass exhibit diminished gut microbiome and fecal butyrate levels, suggesting a potential link between gut microbiome composition and muscle mass rather than muscle function. Certain gut microbes, including Marvinbryantia, Akkermansia, Subdoligranulum, Flavonifractor, and F.prausnitzii (also known as *Faecalibacterium prausnitzii*), have been found to be significantly linked to low skeletal muscle mass in the elderly, providing valuable insights into microbial-mediated pathways contributing to sarcopenia (81). A separate study demonstrated a significant increase in fecal butyrate levels among elderly individuals with preserved skeletal muscle mass (81), reinforcing the association between gut microbiota and skeletal muscle mass. It has been postulated that a transition of the gut microbiota from a protective to a proinflammatory role may disrupt immune response and host metabolism, ultimately resulting in a state of low-grade inflammation that upregulates molecular pathways associated with sarcopenia, ultimately leading to skeletal muscle injury and frailty (91). Nevertheless, insufficient evidence supports the direct impact of the gut microbiome on increasing muscle mass. Further research is warranted to explore the relationship between intestinal microbes and skeletal muscle mass and investigate which metabolites produced by intestinal microbes are linked to skeletal muscle mass.

### 4.5 Improved immune aging through the regulation of intestinal flora imbalance

It has been proposed that maintaining a useful or beneficial gut microbiota structure during the aging process potentially delays or restricts immune senescence (92). As such, regulating immune senescence in elderly individuals by modulating the gut microbiota represents a promising therapeutic approach. For instance, oral supplementation of bifidobacterium can enhance the proportion of lymphocytes (85). Cho et al., in animal experiments, observed that syringaresinol (SYR) could notably increase the Firmicutes/Bacteroides ratio by promoting beneficial bacteria (Lactobacillus and Bifidobacterium) while reducing opportunistic pathogenic bacteria. This alteration in microbial diversity would lead to an increase in total CD3T cells and naive T cells, thereby delaying immune aging (93).

## 5 Conclusion

In conclusion, given the intricate interplay among gut microbiota imbalance, sarcopenia, immune senescence, and T cells, the modulation of gut microbiota holds potential not only for enhancing immune function but also for improving muscle mass and strength. Therefore, can modulating gut microbiota improve muscle mass/strength by bolstering immune function in patients with sarcopenia? Further research is warranted to address this question.

Furthermore, the current understanding of the potential interplay among these three factors and the precise role of T cells in regulating intestinal microbiota in sarcopenia patients still needs to be completed. It remains to be elucidated which specific targets and pathways within these interconnected mechanisms could be leveraged to prevent and ameliorate sarcopenia. Additionally, it is essential to identify pharmaceutical agents that can modulate these targets and pathways to prevent and treat sarcopenia. Furthermore, it is crucial to determine whether these interventions can be effectively translated into clinical practice for predicting, diagnosing, and preventing sarcopenia and developing innovative microbiome-based or immunosenescence interventions for promoting healthy aging. Identifying targets that may lead to therapeutic agents enhancing muscle regeneration and mitigating the impact of muscle wasting during aging and disease is also imperative. Is it feasible to conduct individualized interventions targeting intestinal flora in sarcopenia patients? These are all areas requiring further exploration in future research.

Sarcopenia is a significant global public health concern that damages individual physical function. Currently, the Food and Drug Administration (FDA) has not sanctioned specific medications for sarcopenia treatment, thus necessitating urgent resolution of these issues. Addressing sarcopenia can facilitate the promotion of healthy aging. Presently, there remains a lack of awareness among the elderly regarding sarcopenia. Therefore, communities, hospitals, and society must enhance dissemination and education on knowledge related to sarcopenia so that older individuals can proactively prevent its onset and progression through physical exercise, improved nutrition, and probiotic supplementation. Aging poses a serious challenge in our country, and instilling an ethos of “self-sustained health” in older people can effectively alleviate familial and societal burdens.
